# Business-Oriented Security Analysis of 6G for eHealth: An Impact Assessment Approach

**DOI:** 10.3390/s23094226

**Published:** 2023-04-23

**Authors:** Chiara Suraci, Sara Pizzi, Antonella Molinaro, Giuseppe Araniti

**Affiliations:** Department of Information Engineering, Infrastructure and Sustainable Energy (DIIES), University Mediterranea of Reggio Calabria, 89100 Reggio Calabria, Italy

**Keywords:** eHealth, B5G, 6G, security, business relationships

## Abstract

Following the COVID-19 outbreak, the health sector is undergoing a deep transformation that is increasingly pushing it towards the exploitation of technology, thus fostering the growth of digital health (eHealth). Cellular networks play a pivotal role in promoting the digitalization of healthcare, and researchers are banking on beyond fifth-generation (B5G) and sixth-generation (6G) technologies to reach the turning point, given that, according to forecasts, 5G will not be able to meet future expectations. Security is an aspect that definitely should not be overlooked for the success of eHealth to occur. This work aims to address the security issue from a poorly explored viewpoint, namely that of economics. In this paper, we first describe the main eHealth services, highlighting the key stakeholders involved. Then, we discuss how next-generation technologies could support these services to identify possible business relationships and, therefore, to realize an innovative business-oriented security analysis. A qualitative assessment of the impact of specific security breaches in diverse business conditions is provided. Moreover, we examine a case study in order to show the effects of security attacks in a definite scenario and discuss their impact on business dynamics.

## 1. Introduction

**Figure 1:**
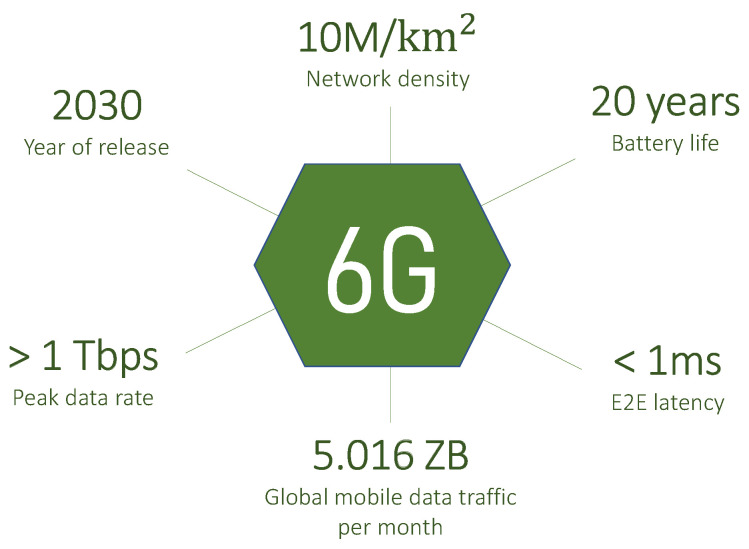
Expected 6G hallmarks.

Although the fifth generation (5G) of mobile networks has not yet spread worldwide, researchers are already working on the specification of the sixth generation (6G). Indeed, the capacity of 5G will not be able to cover the traffic volume per subscription expected for 2030 [1]. Several works in the literature provide overviews of enablers, technologies, applications, and requirements related to 6G [2,3]. These suggest that the features envisaged for 6G will overcome the shortcomings of 5G, to meet the more stringent requirements of the services of the future. To name a few, the 6G peak data rate is expected to exceed 1 Tbps, and end-to-end (E2E) latency to be less than 1 ms [4]; these and other 6G hallmarks are shown in Figure 1.

Along with improved performance, the urgency of ensuring network security has grown increasingly as the cellular network generations evolve. To date, the unstoppable pervasiveness of Information and Communication Technologies (ICT) in manifold fields is undeniable. To cite an example, the success of the digitalization of supply chains has been established for some years, which represents an advantage from many viewpoints, such as maximizing profits, but also a danger from a security perspective because the use of various technologies opens the door to several kinds of cybersecurity attacks [5]. As the authors of [6] explain, this can have an extremely damaging impact on business, potentially causing loss of data and intellectual property, interference in business operations, and reputational harm. In the future society, security must influence business processes, for example, in the assignment of roles and responsibilities, as this could significantly reduce the occurrence of cybersecurity attacks [7]. The security of 5G has become a critical requirement since business dynamics have expanded to actors belonging to disparate fields, thus implying that the set of players who could breach network security is very diversified [8]. 6G is forecast to follow the same techno-economic path as 5G [9]. It is fair to state that the complexity of the 6G business ecosystem will continue to grow because increasingly heterogeneous actors will collaborate to provide cutting-edge services, and business models for telecommunications operators will deeply change due to the revolution that the modalities in providing services will undergo in beyond 5G (B5G) [10]. The security issues that must be addressed to achieve the business-related results expected for 6G are emphasized in [11], where the authors draw attention to the fact that cybersecurity management will be crucial in the hyperconnected world of 2030, as attacks on security could cause disruption or manipulation of technology with severe damage to businesses; this proves the existence of the tie between security and business, as one can impact the other and vice versa. It is needless to specify that the methods used to ensure security will have to be advanced and keep pace with technological progress.

The evolution of mobile security is thoroughly described in the literature [12,13]; to bring forth the need to probe innovative security means to face the challenges expected for 6G. The authors of [14] shed light on the roles of trust, security, and privacy in 6G networks by providing a definition for each concept: *trust* consists in satisfying the expectations of users who fulfill a communication or any other action on a network; *security* is related to the measures implemented to protect applications and must be integrated into the innovative technologies; *privacy* concerns the entities who access users’ personal data and how the data are used. Furthermore, an interesting 6G security-related question is raised in [14], where 6G-enabling technologies are described as worthwhile means of improving network security but, at the same time, possible facilitators of more dangerous and complex attacks. For example, Federated Learning (FL) can promote network security because the data of users are saved locally and only information acquired through distributed learning is then shared [15]. Edge computing and device-to-device (D2D) paradigms can improve network security in future 6G systems, but they could also increase network vulnerabilities by being distributed paradigms [16]. In other words, to fully understand the security issues of 6G, the vulnerabilities of the technologies that will support the forthcoming cutting-edge services must be carefully focused on. For example, the use of platforms and services based on Artificial Intelligence (AI) is strongly promoted by worldwide governments to offer beneficial machines to improve the quality of life of the population; however, the study of countermeasures to AI attacks in 6G is still in its infancy [9]. The use of holography in various sectors, including education, healthcare, and manufacturing, will be widely promoted in 6G networks to shorten distances and enable the provision of highly innovative services; nevertheless, the security and reliability of the network must be significantly improved in order to achieve these goals [17]. The development of 6G could, in general, help to achieve the goal of bringing connectivity to remote areas and improving the quality of life of the people who live there. For example, distance learning and interactive teaching enabled by broadband connections may help the enhancement of education in disadvantaged areas; also in this case, providing security solutions to increase trust in the innovative services provided by 6G and to protect data and users is of paramount importance [18].

The uncontrolled spread of COVID-19 has put the spotlight on the healthcare sector, which, now more than ever, needs an evolution of digitalization that is also characterized by the careful preservation of trust, security, and privacy. The importance of technological support and security measures in resolving the worldwide health emergency is emphasized in [19], where novel smart and connected health solutions to fight COVID-19 are proposed. The security protection of data transmitted for monitoring with connected pacemakers is tested in [20], where the authors aim to prove that patients and healthcare infrastructure can suffer the consequences of new cybersecurity threats when medical devices are connected to the Internet. In [21], we proposed an authentication protocol to protect communications between the resource-constrained Internet of Medical Things (IoMT) nodes that collect health data and the controllers in charge of receiving data from them, intending to introduce a solution for secure and lightweight 6G digital health (eHealth) systems. Indeed, eHealth is considered a 6G use case because 6G is expected to provide eHealth services with the connectivity requirements that they need and to be crucial in the remote management of diagnosis and treatment of diseases [22].

Based on the literature, the telecommunications ecosystem is increasingly business-driven and heavily dependent on trust, security, and privacy for the protection of users and data [23,24]. As also stated in [25], the success of a generation of wireless networks is, to some extent, dictated by an accurate techno-economic assessment, hence, by the analysis of correlations between business policies and strategic telecommunications decisions, including those related to the security of networks.

To the best of our knowledge, the topic of the impact of economic strategies on network security, and vice versa, is not yet sufficiently explored in the literature; in particular, little has been written on the business relationships potentially established among the stakeholders who might work together to provide eHealth services, or on security-related issues. These are the main motivations of this work. In fact, to bridge this gap, this paper provides contributions that can be summarized by the following points:

1. The eHealth use case is placed in the 6G context, and the most significant eHealth services, as identified by the European Commission in [26], are described. A proposal of how key 5G/B5G/6G (hereinafter referred to as “next-generation”) technologies could support these services is provided.
2. Based on the scenarios that can be obtained by applying next-generation technologies to eHealth services, the main involved stakeholders are identified, and possible business relationship models (BRMs) are defined. The meaning of the term BRM to which we refer in this paper will be clarified later on.
3. A qualitative impact estimation is produced for each stakeholder involved in the analyzed BRMs. The impact is assessed by examining the breach of different security requirements, to investigate several attack scenarios. This sort of what-if analysis makes it possible to identify the correlations between business aspects and strategic decisions on the security of cellular networks.
4. A case study is analyzed in order to simulate the occurrence of security attacks under varying levels of danger on the architecture described in [21]. The simulative results illustrate the effects of security violations on the nodes performing the authentication protocol presented in [21] and provide information on the conditions that should alarm the involved stakeholders, therefore suggesting how security attacks could impact business dynamics.

The paper is organized as follows. The eHealth services are described in Section 2, where the mapping between next-generation technologies and services is also provided, in order to illustrate how the former could support the latter. A qualitative business-oriented impact assessment is developed in Section 3, while the effects of the occurrence of malicious attacks in a telemonitoring scenario are shown in Section 4. Conclusions are discussed in Section 5.

## 2. eHealth as a 6G Use Case

The application of ICT to facilitate access to care and improve human health and society’s lifestyle produces the eHealth paradigm. In [27], a link is yielded between the 6G ecosystem and the United Nations’ Sustainable Development Goals (UN SDGs). In particular, the “good health and well-being” SDG strictly depends on ICT, because the evolution of mobile networks represents a key means to reduce the distance between doctors and patients so that the latter can smoothly leverage eHealth services [27]. A description of the services useful for the management of the main remote medicine practices is provided below, followed by a mapping between the next-generation technologies and the described services.

### 2.1. eHealth Services

The eHealth services currently used worldwide are destined to evolve along with the evolution of technologies because, with increasing technical support offered to services, their pervasiveness and functionalities could become greater. The three categories of services referred to in this work capture the majority of proposals in the eHealth literature and are recognized as the most significant by the European Commission [26]. Although 6G may enable numerous other services (some of which will be briefly described later in the section), the methodology used to conduct the qualitative analysis proposed in this work is based on a study of the literature and the results of interviews with medical experts. We have decided not to focus on excessively futuristic services, to have enough literature to analyze, and not to ask experts about services that are too distant from current practices. The services we have decided to consider are not currently exploited to their full potential worldwide. The idea behind this work is that these potentials could be achieved thanks to next-generation technologies.

- *Telemonitoring* enables the continuous collection of medical data through the use of sensors on the patient, whose monitoring can be improved thanks to a more frequent and constant detection of vital parameters. Furthermore, thanks to telemonitoring, the frequency of patient checks held in person can be reduced.
- *Teleconsultation doctor–patient (TDP)* allows a doctor, or a healthcare professional in general, to interact remotely and in real time with a patient. This operating mode cannot replace the first visit, which necessarily requires an in-person meeting between doctor and patient, but it is suitable for the management of checks or all those situations in which physical medical examinations of the patient are not required.
- *Teleconsultation doctor–doctor (TDD)* represents a way to provide a second specialist opinion, as it consists of communication, even asynchronous, between doctors who collaborate in defining the details of a medical report. It requires real-time interaction in emergency cases, e.g., to enable communication between a paramedic and a medical specialist located in two different places.

Alongside these services, telesurgery may be enabled by the ultra-low latency expected for 6G. Furthermore, the massive use of unmanned aerial vehicles (UAVs) in 6G could revolutionize the healthcare sector, making telemedicine services more accessible and facilitating the emergency transfer of, e.g., medicines, blood, organs, after some challenges that currently prevent such applications are solved. A human digital twin paradigm is another forward-looking service realizable with the advent of 6G.

### 2.2. Next-Generation Technologies for eHealth Services

To guarantee unprecedented requirements for services, the confluence of successful past trends and emerging trends will have a vital role [28]. In this section, we discuss how technologies considered crucial for 5G/B5G/6G [29], referred to as next-generation technologies, could lead to a game-changing realization of eHealth services. Many of the technologies that will be mentioned throughout this section could greatly benefit from the use of cloud systems, both centralized and distributed, the latter being often preferred in reference to future 6G networks. For example, the use of Multi-access Edge Computing (MEC) servers, bringing cloud resources closer to the user, would be able to guarantee the optimization of critical parameters in eHealth services, such as latency and energy saving.

The telemonitoring service requires sensors distributed on a patient to detect and transmit data to the doctor, who can thus monitor health conditions and the progress of any chronic diseases. *Internet of Everything (IoE)* represents the most suitable paradigm for efficient management of the telemonitoring service, as it consists of the evolution of the Internet of Things (IoT) to encompass sensing devices related to everything (e.g., objects, people). The most disparate parameters are expected to be collected thanks to IoE, such as bio-signals, temperature, and pressure, guaranteeing requirements for high data rate, high scalability, and low latency. Although the demands of the telemonitoring service can already be satisfied by exploiting the IoT paradigm on 5G systems, the integration of 6G and IoE would allow monitoring processes to be improved by the use of advanced and highly-performing devices. *AI*, which is considered one of the most powerful and characteristic technologies of the future 6G, could ensure optimal management of the telemonitoring service by enabling the extraction of valuable information from data measured by sensors distributed on patients. The result of applying AI to the health sector is also known as Intelligent Healthcare or Healthcare 5.0, and will benefit from the support of 6G networks as it is expected that it will require a data rate above 24 Gbps. Softwarization and virtualization are equally useful techniques in effectively managing telemonitoring. The *Software-Defined Networking (SDN)* approach, allowing the centralization of control of network devices (e.g., routers, switches), could optimize the data collection process. *Network Slicing* could be a valuable means for the separate management of monitoring related to different patients. The full deployment of the *Digital Twin* paradigm is expected to be achieved with 6G. It is a dynamic virtual representation of an entity, continuously updated to provide an accurate real-time status of the physical twin. One of its main applications consists of the predictive maintenance of IoT devices used, for example, in Industry 4.0. If applied to the telemonitoring service, it could guarantee numerous other advantages. For example, relying on the continuous and promising progress of technologies used in the medical field, the digital twin could be exploited to replicate some organs of the human body, or even the entire body of the patient, to personalize treatments or to predict the effects of a disease before their manifestations. In summary, the main technologies useful for telemonitoring are IoE, AI, SDN, Network Slicing, and Digital Twin.

Differently from telemonitoring, the TDP service asks for the support of technologies able to enable reliable real-time communications; furthermore, the doctor requires the means to remotely visit the patient as well if possible. *Holographic-Type Communications (HTC)*, *Extended Reality (XR)*, and *Multi-Sense Experience (MSE)* are distinctive trends of the future 6G and represent ideal solutions for the doctor to have a complete and immersive experience with the patient. Thanks to HTC, patients could be projected in doctors’ offices as high-definition holograms, thus allowing the doctors to have a complete view of their physical characteristics. Similarly, the wearables used for the XR experience could provide doctors with patients’ details for the teleconsultation experience. Thanks to the implementation of the MSE, teleconsultation might not be limited to a simple visual or acoustic interaction; alternatively, it could allow the transmission of information receivable with smell or taste, which would make the TDP an experience close to the in-person medical visit. All of these technologies require the fulfillment of strict connectivity, data rate, and latency requirements; therefore, the advent of 6G is necessary for them to be widely used. For example, millimeter waves leveraged by 5G may not be sufficient to meet the requirements of these technologies that, instead, could rely on Terahertz (THz) waves, expected to be explored with 6G. In summary, the main technologies useful for TDP are HTC, XR, and MSE.

Regarding the TDD service, overall, it could take advantage of the same technologies described for TDP, with the addition of some others that could enable other important features. In particular, *Tactile Internet* could be exploited if TDD is performed for remote surgery, while *On-Board Communications (OBC)* could help in the management of TDD involving paramedics in ambulances. In summary, the main technologies useful for TDD are the same as TDP (i.e., HTC, XR, MSE), with the addition of Tactile Internet and OBC.

## 3. Business-Oriented Security Analysis

In this section, we describe possible BRMs among the stakeholders involved in the application of next-generation technologies to eHealth services, as described in Section 2.2. The considered stakeholders are inspired by [27], where the 6G key players are listed. For the purposes of this work, we use the term *business relationship model* to indicate the relations that are established between the actors involved in the provision of resources necessary for the delivery of a service, also functional to the definition of business models. In fact, a business model consists in the strategies implemented by the actors of a company to offer a product and profit from it [30]. The introduced BRMs are built based on the considered next-generation technology to highlight the effect that the implementation of forthcoming technologies can have on business dynamics and, consequently, the heterogeneity that will characterize the stakeholders’ ecosystem of future 6G networks. In other words, the technology defines the BRM that identifies the actors possibly involved in the analyzed service and for which the potential expected damage assessment is carried out. Mainly, vertical relations involving no more than four actors are considered in our work: actor $a_{1}$ has a business relationship with $a_{2}$, who has a business relationship with $a_{3}$, and so on. More complex scenarios are likely to be possible; however, many of them can be modeled based on our proposals.

Table 1, Table 2 and Table 3 report a qualitative impact assessment for the BRMs of each service. The *potential expected damage* is indicated for each involved stakeholder, based on the impact that the violation of a given security requirement would have on the affected actor for the considered BRM: for example, a breach of the $r_{1}$ requirement for actor $a_{1}$ has a high impact when $a_{1}$ takes the most damage in the provision of the service in question. The methodology used to evaluate the potential expected damage is inspired by the one applied in [8] and consists of a qualitative estimation of the impact based on the literature and interviews with medical experts, conducted as part of the iCare project (funded within POR FESR FSE 2014/2020 of the Calabria region, with the participation of European Community Resources of FESR and FSE, of Italy and Calabria). The literature review allowed us to identify the most frequent attacks, the major security requirements, and the most vulnerable assets in the eHealth field. The information collected was enriched with the results obtained from interviews with hospital doctors from different sectors. In particular, these interviews were helpful because they allowed us to conduct a sort of cost-benefit analysis that considers the security assurance of the most vulnerable assets in the application of eHealth services. The estimated impact can take a value of HIGH, MEDIUM, or LOW, in line with the approach typically used in a qualitative risk analysis [31], which we deem the most appropriate for our primary goal of providing a general-purpose impact assessment. It is worth mentioning that the proposed analysis refers to the techno-economic ecosystem expected for 6G, but is not dependent on any Radio Access Network (RAN) technology because there is still no specification for 6G.

The investigated security requirements are: *(i) authentication*, meaning mutual authentication between stakeholders, a violation of which would result in the theft and misuse of the credentials of some actors; *(ii) data protection*, which concerns the confidentiality and integrity of data; *(iii) privacy*, related to identity privacy protection; *(iv) resilience*, intended as the ability of the system on which the service relies most to work even after the occurrence of adversity.

**Figure 2:**
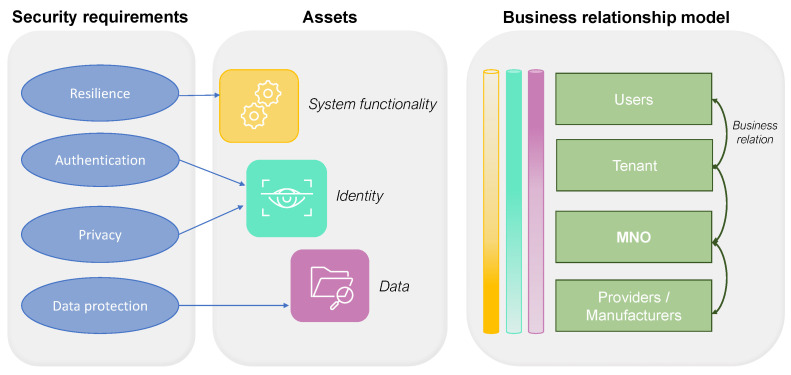
A general scheme including security requirements, assets, and BRM; it illustrates which asset each requirement refers to and the level of importance of the diverse assets for each stakeholder (indicated by the color gradient in the cylinders, where more color means more importance).

Figure 2 illustrates the logic behind the business-oriented security analysis; it consists of a general scheme detailed below for each analyzed service.

### 3.1. Telemonitoring

#### 3.1.1. BRM

Manufacturers/Developers, Mobile Network Operator (MNO), Tenant (i.e., the hospital institution that requires connectivity services to the MNO to provide eHealth services), and Users (i.e., doctors and patients who benefit from the offered services) are the stakeholders possibly involved in the BRM that could be determined by the use of IoE, AI, SDN, and Digital Twin to support the telemonitoring service. The BRM investigated for Network Slicing is not entirely based on vertical relationships, because we want to highlight the risk deriving from an attack that penetrates the “horizontal” separation between resources related to two diverse patients: MNO has a vertical relationship with Tenant, who has the same type of business relationship with both User 1 and User 2.

#### 3.1.2. Impact Assessment

Examining Table 1, the damage caused by a possible violation of the authentication requirement is high for tenant and users, medium for MNO, and low for IoE equipment manufacturers. To properly interpret these results, it is necessary to relate them to the considered scenario, i.e., the delivery of the telemonitoring service through the IoE. To provide an example, we can imagine a violation of mutual authentication between tenant and users, such as in the case of the occurrence of a brute-force attack that would allow the attacker to obtain the tenant’s credentials, thus deceiving the authenticated users: to whom would this action cause the most damage? Given the context, certainly to the hospital (tenant), whose credential theft could provoke the disclosure of highly sensitive information, and to doctors and patients (users), who could risk exchanging confidential information with a malicious attacker. We can see eye-to-eye for data protection, a breach of which would cause high damage for tenant and users, medium for MNO, and low for IoE equipment manufacturers. Privacy protection is critical for doctors and patients. For the resilience requirement, a slightly different analysis should be applied because it must be seen as a system parameter; therefore, a lack of resilience produces greater damage to IoE equipment manufacturers and MNO, mainly responsible for managing the service.

In the case of telemonitoring supported by AI, compared with the previous, the considered scenario requires less involvement of the network for the success of the service, which mainly relies on AI software developers: in other words, the secure transmission of data across the network is a crucial aspect of the IoE paradigm, while, when it comes to AI, the critical point is the software module that performs the operations. This is why MNO is less exposed to the damage caused by any attacks on authentication, data, or resilience, while AI Software Developers are the main victims of attacks on resilience. The authors of [32] provide some examples of machine learning (ML) attacks that can cause violations of the data protection requirement, thus causing greater damage to tenants and users, especially in the case of highly sensitive and significant data, such as healthcare-related ones. For example, Poisoning Attacks could lead to the manipulation of data collected for ML, resulting in serious consequences for doctors and patients.

We assume that SDN controllers are managed by the MNO, and that network devices are built and supervised by external manufacturers. In light of this, the most eye-catching result in the table is related to resilience, a violation of which would cause high damage to the actor who controls the network (i.e., MNO), and medium damage to those responsible for network equipment (i.e., network equipment manufacturers). Denial of service (DoS) is an example of a frequent attack on the resilience of SDN networks that, by making network management difficult, would cause the greatest damage to the MNO [33].

The results linked to the Network Slicing case should be interpreted based on the business relations described above. The main security vulnerabilities of network slicing relate to resource isolation [34]. For example, an attacker could penetrate the interslice separation between Users 1 and 2, whose slices share the same physical infrastructure provided by an MNO, thus causing a violation of data belonging to the users. Another example could be the occurrence of an impersonation attack that manages to breach the mutual authentication between the MNO and tenants: in this case, the tenants would be exposed to high risks, but the MNO could also suffer medium damage, which can be estimated as slightly lower than that caused to tenants due to the lower sensitivity of network data compared with health data [35].

### 3.2. Teleconsultation Doctor–Patient

#### 3.2.1. BRM

A general BRM can be hypothesized to define the relationships among the stakeholders involved in the provision of the TDP service supported by the technologies previously described. For HTC, XR, and MSE, it can be assumed that: the sensors used to collect inputs on a patient are produced by specific manufacturers; the inputs collected by the sensors are processed by applications offered by specific developers; the results of the applications travel through the network managed by the MNO; doctors and patients, through the tenant, benefit from the results of the elaborations.

#### 3.2.2. Impact Assessment

Regarding Table 2, hospitals, doctors and patients could suffer the most damage from an authentication violation. The same applies to data protection, as the data of tenants and users are the most attractive to attackers; network data belonging to the MNO could suffer medium damage, because they are less sensitive than health data. Identity privacy protection is critical for tenants and users. Concerning the resilience requirement, application developers are primarily responsible for the execution of the service; thus, they could suffer the highest damage.

### 3.3. Teleconsultation Doctor–Doctor

#### 3.3.1. BRM

In the case of Tactile Internet and OBC, which should both rely on extremely low end-to-end latency, very high data rates, and efficient mobility management, the network is the main entity responsible for the good functioning of the TDD. This is why Table 3 presents a BRM focused on the stakeholders possibly involved in network management. In particular, the network infrastructure is assumed to be managed by an entity (i.e., Infrastructure Provider) other than the one that provides mobile connectivity services (i.e., Mobile Service Provider (MSP)).

#### 3.3.2. Impact Assessment

The results presented in Table 3 can be interpreted based on the described BRM and following the same logic as the other services. Therefore, a possible theft of credentials resulting in a violation of the authentication requirement could cause high damage to tenants and users, medium damage to MSP, and low damage to infrastructure providers. The most sensitive (i.e., vulnerable) data belong to tenants and users, the primary victims of attacks on data. The protection of users’ privacy is definitely the most critical consideration in the provision of a TDD service that exploits, for example, the OBC to optimize remote medical assistance practices in emergencies. Finally, the infrastructure provider and the MSP would suffer the highest damage in the event of attacks on the resilience of the system leveraged by the TDD service.

## 4. Case Study: How Can Security Attacks Impact Business Dynamics?

In Section 3, the impact is qualitatively estimated under fixed attack conditions but varying the business dynamics established for each service based on the implemented technologies; in this section, a service with a fixed BRM is investigated by varying the attack conditions, to illustrate the effects caused by diverse threats. The architecture under analysis is the one presented in [21], which consists of three layers for the sensing, processing, and storage of health data detected on patients. In particular: *sensors* belong to the lowest layer of the architecture, as they are deployed for data detection; similar to an SDN solution, *controllers* are distinct physical nodes deployed to instruct a certain number of sensors, clustered according to the patient on whom they are installed, and to collect and process data from them; MEC servers represent the entity primarily delegated to data storage. Regarding the considered BRM, we refer to the SDN case reported in Table 1 for the telemonitoring service: sensors are managed by external manufacturers, while controllers and MEC servers are managed by the MNO. Therefore, sensors and controllers are deployed by different stakeholders for diverse scopes. We assume that they could be malicious, intentionally or because they are victims of attacks, thus breaching the authentication protocol performed according to the procedures described in [21]. A malicious sensor could be able to overcome the physical separation from its controller by exploiting the functional link between them, thus damaging it and the assets of the stakeholder who handles it. Conversely, if a controller is malicious, the sensors it manages and the information they send to the controller are in danger.

**Figure 3:**
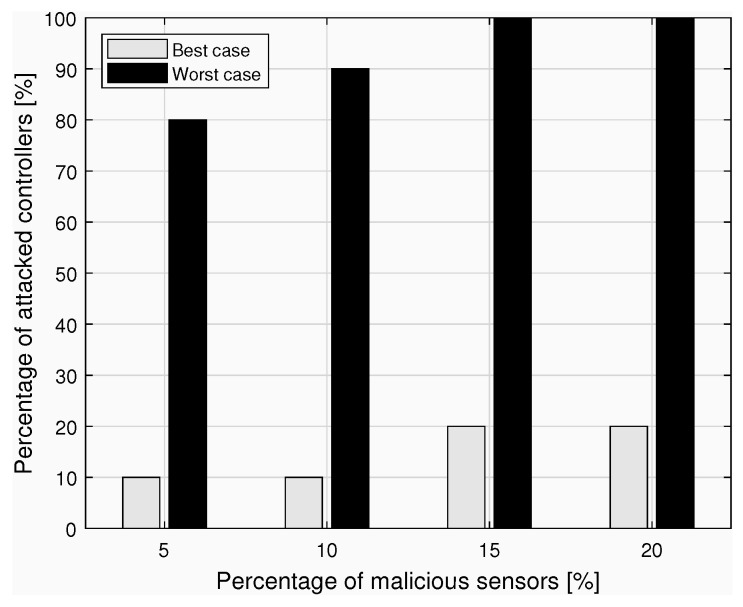
The effects of attacks by malicious sensors.

In Figure 3, the trend of the percentage of controllers attacked by the malicious sensors is shown in two cases: in the *best case*, malicious sensors are concentrated in the fewest possible number of controller nodes; in the *worst case*, the percentage of malicious sensors is spread over several controllers deployed in the network.

**Figure 4:**
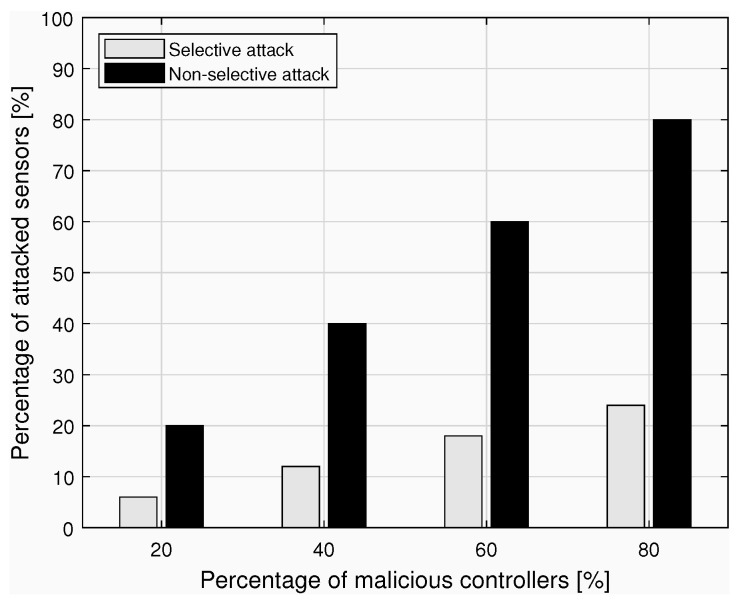
The effects of selective and non-selective attacks by malicious controllers.

Figure 4 is dually obtained by considering an increasing percentage of malicious controllers to show the percentage of sensors managed by the malicious controller and actually attacked. The two cases compared are: *selective*, where the attack profile implemented by the malicious controller does not affect all the controlled sensors; and *non-selective*, where the malicious controller attacks all the managed sensors.

In summary, we have applied one of the BRMs defined in Section 3 to a specific architecture and illustrated the effects of possible breaches on the authentication protocol. The aim is to demonstrate how risky the occurrence of certain attacks could be for business, especially when the involved nodes are managed by distinct stakeholders. Based on the qualitative analysis conducted in Section 3 and on an estimate of the occurrence probabilities of the security attacks discussed above, stakeholders are provided with a means to perform a careful risk quantification that can guide adequate investments in security measures.

## 5. Conclusions

The worldwide spread of COVID-19 has revealed two fundamental trends for the development of the society of the future: the increasing value of digitalization in various areas, including the healthcare sector, and the growing importance of economic strategies in fostering technological progress. The advances made in the field of wireless cellular networks will soon lead to the sixth generation, already under investigation by researchers. The complete deployment of 5G in markets around the world and the advent of 6G will represent opportunities to reach a turning point in the digitalization process of the healthcare sector, the shortcomings of which have been brought to light by the needs arising from the health emergency caused by COVID-19. However, the evolution of the digital health paradigm must be accompanied by the improvement of security and privacy mechanisms to be implemented for the protection of data and users.

The main purpose of this work is to provide a correlation between business aspects and security-related strategies in cellular networks supporting eHealth services to make the service provision consistent with the trends and expectations foreseen for 6G networks. In detail, a business-oriented security analysis is accomplished to provide an impact assessment for each stakeholder involved in the introduced BRMs when different security requirements are breached. To obtain plausible BRMs, potential health scenarios have been obtained, assuming the application of next-generation technologies to those services identified by the European Commission as the most widespread eHealth services. The outcome of the performed qualitative analysis works as a tool for the stakeholders to determine the potential expected damage that the exploitation of 6G technologies to eHealth services could bring, and thus, to determine the possible points of failure that deserve particular attention and require specific countermeasures to be put in place. The analysis that we have carried out could help the actors involved in the provision of eHealth services to understand the level of digital trust necessary for specific business dynamics to minimize the risk of cyber attacks. Moreover, stakeholders could understand which aspects to pay more attention to in drafting Service Level Agreements (SLA) that will sanction the contractual obligations between parties.

Furthermore, the effects of concrete security violations are revealed in graphs portraying increasing percentages of malicious network nodes and diverse attack conditions. In so doing, we can demonstrate how central the role of security must be in the digitalization process of the healthcare sector, and how future business dynamics may impact some technological choices aimed at protecting the network and vice versa. 

## Figures and Tables

**Table 1:** Potential expected damage for telemonitoring.

		Authentication	Data Protection	Privacy	Resilience
**IoE**	IoE Equipment Manufacturers	LOW	LOW	LOW	HIGH
	MNO	MEDIUM	MEDIUM	LOW	HIGH
	Tenant	HIGH	HIGH	MEDIUM	LOW
	Users	HIGH	HIGH	HIGH	LOW
**AI**	AI Software Developers	LOW	LOW	LOW	HIGH
	MNO	LOW	LOW	LOW	LOW
	Tenant	HIGH	HIGH	MEDIUM	LOW
	Users	HIGH	HIGH	HIGH	LOW
**SDN**	Network Equipment Manufacturers	LOW	LOW	LOW	MEDIUM
	MNO	MEDIUM	MEDIUM	LOW	HIGH
	Tenant	HIGH	HIGH	MEDIUM	LOW
	Users	HIGH	HIGH	HIGH	LOW
**Network Slicing**	MNO	MEDIUM	MEDIUM	LOW	HIGH
	Tenant	HIGH	HIGH	MEDIUM	LOW
	User 1	HIGH	HIGH	HIGH	LOW
	User 2	HIGH	HIGH	HIGH	LOW
**Digital Twin**	Software Developers	LOW	LOW	LOW	HIGH
	MNO	MEDIUM	LOW	LOW	HIGH
	Tenant	HIGH	HIGH	MEDIUM	LOW
	Users	HIGH	HIGH	HIGH	LOW

**Table 2:** Potential expected damage for teleconsultation doctor–patient (TDP).

		Authentication	Data Protection	Privacy	Resilience
**HTC/XR/MSE**	Sensor Equipment Manufacturers	LOW	LOW	LOW	MEDIUM
	Application Developers	LOW	LOW	LOW	HIGH
	MNO	MEDIUM	MEDIUM	LOW	MEDIUM
	Tenant and Users	HIGH	HIGH	HIGH	LOW

**Table 3:** Potential expected damage for teleconsultation doctor–doctor (TDD).

		Authentication	Data Protection	Privacy	Resilience
**Tactile Internet/OBC**	Infrastructure Provider	LOW	LOW	LOW	HIGH
	MSP	MEDIUM	MEDIUM	LOW	HIGH
	Tenant	HIGH	HIGH	MEDIUM	LOW
	Users	HIGH	HIGH	HIGH	LOW

## Data Availability

Not applicable.
